# An Innovative Technique For Extracorporeal Carbon Dioxide Removal Featuring An Electrodialysis Unit: An In-Vitro Experiment

**DOI:** 10.1186/2197-425X-3-S1-A501

**Published:** 2015-10-01

**Authors:** A Zanella, D Ferlicca, S Abd El Aziz El Sayed Deab, S Colombo, S Spina, S Sosio, M Introna, D Ceriani, D Salerno, A Pesenti

**Affiliations:** Università degli Studi di Milano Bicocca, Monza, Italy

## Introduction

Acidification of blood entering the membrane lung (ML) converts bicarbonate ions into dissolved gaseous CO_2_, increasing the pCO_2_ transmembrane gradient and thus the extracorporeal carbon dioxide removal (ECCO_2_R) [1]. Extracorporeal blood acidification has previously been achieved by infusion of lactic acid, which proved to be effective in rising ECCO_2_R but determined a mild increase of total CO_2_ production and induced a slight degree of metabolic acidosis [2], thus limiting the overall effectiveness of such treatment.

## Objectives

The aim of this study is to evaluate in-vitro the efficiency of an ECCO_2_R technique enhanced by an innovative acidification system featuring an electrodialysis unit, which does not require the infusion of any exogenous acid.

## Methods

The circuit used for this experiment included a *bloodcircuit*, including a dialyzer, and a *dialysiscircuit,* a closed loop circuit featuring an electrodialysis (ED) cell and an adult polypropylene membrane lung (Quadrox-i, Maquet). An aqueous polyelectrolyte carbonated solution (CB 32, Novaselect, pH 7.33 ± 0.02, HCO_3_^-^ 32 mmol/l) was used as a substitute for blood and flowed into the hemofilter at 250 ml/min. the ED unit is able to transfer electrolytes across a semipermeable membrane proportionally to the applied amperage. the ED cell was therefore used to increase chloride concentrations in the dialysate, thus reducing pH before the membrane lung without infusing of any exogenous compound. Five different amperages (0, 2, 4, 6 and 8 Amp) were tested. At the end of each step samples were withdrawn from *blood* and *dialysiscircuit,* and CO_2_ removal (VCO_2_) was measured.

## Results

**Figure 1:**
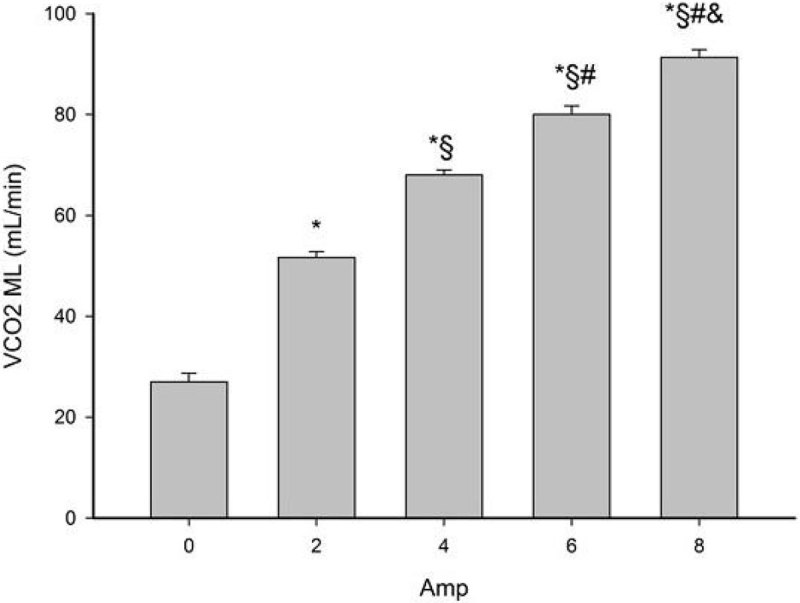
***Membrane lung carbon dioxide removal (VCO***_***2***_ ML) at increasing amperages. *: p < 0.001 vs 0 Amp, §: p < 0.001 vs 2 Amp, #: p < 0.001 vs 4 Amp, &: p < 0.001 vs 6 Amp

The application of ED technique determined an increase in chloride concentration before the ML up to 7.1 ± 1 mEq/L (at 8 Amp) and a consequent reduction in pH from 7.48 ± 0.01 to 6.5 ± 0.04. This resulted in a significant raise of CO_2_ extraction, up to a VCO_2_ increase of 237% at 8 Amp, (see Figure 1).

## Conclusions

The tested prototype ECCO_2_R device, enhanced by an electrodialysis unit, proved to be effective in increasing carbon dioxide removal, proportionally to the applied amperage. Future experimental studies are required to evaluate in-vivo this innovative technique.
